# LASSO-based machine learning models for the prediction of central lymph node metastasis in clinically negative patients with papillary thyroid carcinoma

**DOI:** 10.3389/fendo.2022.1030045

**Published:** 2022-11-23

**Authors:** Jia-Wei Feng, Jing Ye, Gao-Feng Qi, Li-Zhao Hong, Fei Wang, Sheng-Yong Liu, Yong Jiang

**Affiliations:** The Third Affiliated Hospital of Soochow University, Changzhou First People’s Hospital, Changzhou, Jiangsu, China

**Keywords:** papillary thyroid carcinoma, central lymph node metastasis, machine learning, prediction model, random forest

## Abstract

**Background:**

The presence of central lymph node metastasis (CLNM) is crucial for surgical decision-making in clinical N0 (cN0) papillary thyroid carcinoma (PTC) patients. We aimed to develop and validate machine learning (ML) algorithms-based models for predicting the risk of CLNM in cN0 patients.

**Methods:**

A total of 1099 PTC patients with cN0 central neck from July 2019 to March 2022 at our institution were retrospectively analyzed. All patients were randomly split into the training dataset (70%) and the validation dataset (30%). Eight ML algorithms, including the Logistic Regression, Gradient Boosting Machine, Extreme Gradient Boosting (XGB), Random Forest (RF), Decision Tree, Neural Network, Support Vector Machine and Bayesian Network were used to evaluate the risk of CLNM. The performance of ML models was evaluated by the area under curve (AUC), sensitivity, specificity, and decision curve analysis (DCA).

**Results:**

We firstly used the LASSO Logistic regression method to select the most relevant factors for predicting CLNM. The AUC of XGB was slightly higher than RF (0.907 and 0.902, respectively). According to DCA, RF model significantly outperformed XGB model at most threshold points and was therefore used to develop the predictive model. The diagnostic performance of RF algorithm was dependent on the following nine top-rank variables: size, margin, extrathyroidal extension, sex, echogenic foci, shape, number, lateral lymph node metastasis and chronic lymphocytic thyroiditis.

**Conclusion:**

By incorporating clinicopathological and sonographic characteristics, we developed ML-based models, suggesting that this non-invasive method can be applied to facilitate individualized prediction of occult CLNM in cN0 central neck PTC patients.

## Introduction

Thyroid papillary carcinoma (PTC), the most common histological type of thyroid cancer, has been increasing rapidly (1). The incidence of lymph node metastasis (LNM) is high, ranging from 49% to 90% (2, 3). Central compartment is the first area for the metastasis of PTC. This area extends from the inferior border of the hyoid bone to the superior border of the sternum and is bilaterally bounded by the bilateral common carotid arteries. According to previous studies, PTC patients with central lymph node metastasis (CLNM) have an increased risk of regional recurrence (4, 5).

It is still controversial whether clinically negative (cN0) central neck patients should routinely perform preventive central node dissection (CND). Guidelines from China and Japan are more aggressive, and they suggest that prophylactic CND should be routinely performed with appropriate protection of the parathyroid glands and recurrent laryngeal nerve (6, 7). Conversely, for T1 or T2, non-invasive and cN0 PTC patients, the American Thyroid Association (ATA) guidelines do not recommend prophylactic CND (8). Therefore, the status of the central lymph nodes is crucial for the management of PTC patients, especially the decision-making of surgical methods.

Currently, high-resolution ultrasound is still the first choice for preoperative evaluation of cervical lymph nodes in patients with PTC. However, its sensitivity is low, resulting in some false-negative rates. As reported, the diagnostic sensitivity of ultrasound to cervical LNM is only about 20% to 40% (9, 10). Hence, occult LNM has been reported to occur in about 27% to 55% of PTC patients with cN0 neck (11, 12).

Although the risk factors of CLNM have been reported and several prediction models have been established, these results are inconsistent. This is mainly due t o the complexity of medical data, and there are significant differences in the calculation methods of the model. Therefore, we intend to use a new type of artificial intelligence, namely machine learning (ML), to analyze the connections between important data and make accurate decisions (13–17).

By using clinical and sonographical characteristics associated with CLNM, we aimed to develop models based on eight ML algorithms to predict CLNM in patients with cN0 central neck. And then, by selecting one model that performs best in predicting the risk of CLNM, personal strategies could be proposed to help clinicians to make therapeutic decisions.

## Materials and methods

### Study population

This retrospective study was approved by the Ethics Committee of Changzhou First People’s Hospital, and written informed consent was obtained from all patients. Consecutive patients who underwent initial thyroid surgery at our institution between July 2019 and March 2022 were retrospectively reviewed. Exclusion criteria were as follows: (1) non-PTCs or other subtypes than classic PTC; (2) preoperative ultrasound suspected CLNM; (3) history of prior treatment for head and neck cancer; (4) history of cervical radiation exposure in childhood; (5) family history of thyroid cancer; (6) history with other malignancy; (7) incomplete clinical data; (8) loss to follow-up; (9) patients who underwent non-curative surgery (residual tumor or lymph node detected within 6 months of initial surgery). A total of 1099 patients were enrolled in this study.

### Surgical strategy

All patients were treated for thyroid nodules and confirmed as Bethesda Categories V or VI based on ultrasound-guided fine needle aspiration cytology (FNAC). Cervical lymph nodes with the following characteristics were suspected of metastases: hyperechoic changes, roundness or necrosis, loss of the fatty hilum, microcalcification or peripheral vascularity (18). FNAC was performed preoperatively to confirm the histopathological diagnosis of suspicious lateral lymph nodes.

All patients underwent total thyroidectomy or thyroid lobectomy. According to the Chinese guidelines for diagnosis and treatment of differentiated thyroid carcinoma, on the premise of effectively protecting the parathyroid gland and recurrent laryngeal nerve, CND is routinely performed for PTC patients, even in patients with cN0 central neck. Ipsilateral CND is performed for ipsilateral lesion; bilateral CND is performed for isthmus lesion and bilateral lesions. According to the ATA guidelines (8) and Chinese guidelines, lateral neck dissection (LND) is not recommended for patients with cN0 lateral neck. In our institution, LND was performed only in patients with high suspicion of lateral lymph node metastasis (LLNM) based on preoperative imaging data and FNAC.

### Clinical characteristics and sonographical features

A total of 17 variables were analyzed in this study. Clinicopathological characteristics included sex, age, body mass index (BMI), diabetes, BRAF V600E mutation, chronic lymphocytic thyroiditis (CLT), maximum tumor size, the number of foci, bilaterality, location, extrathyroidal extension (ETE) detected during surgery, and LLNM. BMI (kg/m^2^) was defined as weight (kg) divided by height (m) squared. According to the World Health Organization-BMI standard, enrolled PTC patients were divided into normal (BMI < 25 kg/m^2^), overweight (25 ≤ BMI < 30 kg/m^2^), and obese (BMI ≥ 30 kg/m^2^) group. The diagnosis of CLT included any of the following: (i) elevated antibodies to thyroid peroxidase level (>50 IU/mL), and/or (ii) findings of diffuse heterogeneity on ultrasound, and/or (iii) diffuse lymphocytic thyroiditis on histopathology (19). ETE detected during surgery was defined as the primary tumor extending through the thyroid capsule to perithyroidal soft tissue such as perithyroidal fat, or involving strap muscles, or extending to surrounding structures such as larynx, trachea, esophagus, recurrent laryngeal nerve, subcutaneous soft tissue, skin, internal jugular vein, or carotid artery (20).

Preoperative sonographical characteristics of each nodule included the following features: nodular composition, echogenicity, echogenic foci (calcification), shape (aspect ratio) and margin (including irregular shape and ETE). ETE detected by ultrasound was defined as a tumor with capsular abutment of more than 25% of its perimeter on ultrasound (21). More than two radiologists with 10 years of experience in thyroid cancer ultrasound diagnosis evaluated the ultrasound images.

### Feature selection

**Figure 1:**
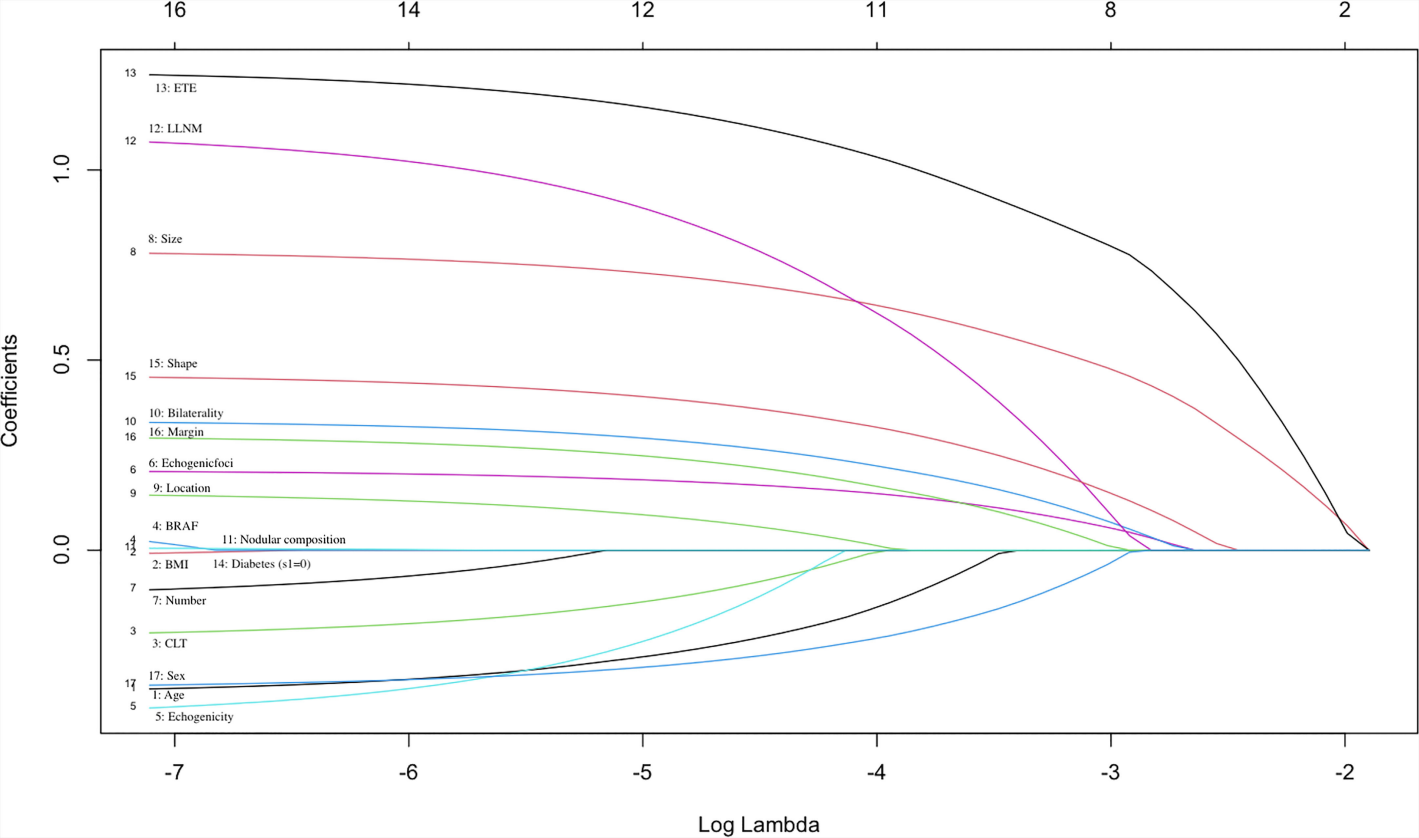
Selection of significant parameters in clinicopathologic variables in the training set. The values of the coefficients and the corresponding lambda values, each curve represents each feature in the model.

The datasets were randomly assigned 70% of datasets to the training set (769 patients) and 30% of datasets to the validation set (330 patients). Feature selection plays an important role in reducing computational complexity and improving classification accuracy. We used the LASSO Logistic regression method to select the best predictive features from the 17 features mentioned above, and finally got the 13 features that were most relevant for predicting CLNM (**Figure 1**).

### Construction, validation, and performance of ML-based models

Eight ML algorithms, including Logistic Regression (LR), Gradient Boosting Machine (GBM), Extreme Gradient Boosting (XGB), Random Forest (RF), Decision Tree (DT), Neural Network (NNET), Support Vector Machine (SVM) and Bayesian Network (BN) were applied in this study (16, 17, 22–24).

By using the same thresholds determined in the training set, we further tested the predictive performance of eight models in the independent validation set. We adopted 10-fold cross-validation method to minimize the adverse effect of overfitting and verify the accuracy of the models. The predictive performance of the above models was assessed by the receiver operating characteristic (ROC) curve and area under the curve (AUC). The closer the AUC was to 1, the better performance of the model. The sensitivity and specificity of the above models were also calculated. Additionally, we employed decision curve analysis (DCA) to assess the clinical utility of the above models s (25).

### Statistical analysis

All statistical analysis was performed by using SPSS Version 25.0 software (Chicago, IL, USA), and R software Version 3.5.3 (The R Foundation for Statistical Computing). Pearson Chi-square test or Fisher’s exact test was used for categorical data. Normally distributed quantitative parameters were compared by Student’s t-test, while non- normally distributed parameters were compared by the Mann-Whitney U test. A *P* value < 0.05 was considered statistically significant. R software (Version 3.5.3) was used to develop ML-based models and DCA.

## Results

### Demographics and sonographic features of PTC patients

**Table 1** shows the clinical and sonographic characteristics of the PTC patients in the training set, validation set, CLNM-positive and CLNM-negative group in the training set. The 1099 patients were divided into two groups randomly: approximately 769 (70%) cases were conducted as the training set, and the remaining around 330 (30%) cases were used as the validation set. There were no significant differences in clinicopathological and sonographic features between the training set and the validation set (*P >*0.05 for all comparisons), justifying their use as training and validation cohorts.

**Table 1:** Clinical and ultrasonic characteristics of the PTC patients.

Characteristics	Training set				Validation set (n = 330)	*P* value*
	Total (n = 769)	CLNM+(n = 389)	CLNM−(n = 380)	*P* value	
Sex						
Male	246 (32.0%)	151 (38.8%)	95 (25.0%)		100 (30.3%)	
Female	523 (68.0%)	238 (61.2%)	285 (75.0%)	<0.001	230 (69.7%)	0.581
Age (Y)						
≥55	142 (18.5%)	62 (15.9%)	80 (21.1%)		64 (19.4%)	
<55	627 (81.5%)	327 (84.1%)	300 (78.9%)	0.068	266 (80.6%)	0.718
BMI (kg/m^2^)						
Normal	32 (4.2%)	21 (5.4%)	11 (2.9%)		12 (3.6%)	
Overweight	467 (60.7%)	225 (57.8%)	242 (63.7%)		207 (62.7%)	
Obesity	270 (35.1%)	143 (36.8%)	127 (33.4%)	0.101	111 (33.6%)	0.797
Diabetes						
Absence	677 (88.0%)	349 (89.7%)	328 (86.3%)		288 (87.3%)	
Presence	92 (12.0%)	40 (10.3%)	52 (13.7%)	0.146	42 (12.7%)	0.723
BRAF V600E mutation						
Negative	85 (11.1%)	43 (11.1%)	42 (11.1%)		41 (12.4%)	
Positive	684 (88.9%)	346 (88.9%)	338 (88.9%)	1.000	289 (87.6%)	0.513
CLT						
Presence	234 (30.4%)	112 (28.8%)	122 (32.1%)		118 (35.8%)	
Absence	535 (69.6%)	277 (71.2%)	258 (67.9%)	0.318	212 (64.2%)	0.083
Maximum tumor size (cm)						
≤1	470 (61.1%)	176 (45.2%)	294 (77.4%)		208 (63.0%)	
>1 to ≤2	201 (26.1%)	141 (36.2%)	60 (15.8%)		87 (26.4%)	
>2 to ≤4	80 (10.4%)	58 (14.9%)	22 (5.8%)		28 (8.5%)	
>4	18 (2.3%)	14 (3.6%)	4 (1.1%)	<0.001	7 (2.1%)	0.788
The number of foci						
1	513 (66.7%)	226 (58.1%)	287 (75.5%)		230 (69.7%)	
2	188 (24.4%)	123 (31.6%)	65 (17.1%)		72 (21.8%)	
3 or more	68 (8.8%)	40 (10.3%)	28 (7.4%)	<0.001	28 (8.5%)	0.603
Bilaterality						
Absence	607 (78.9%)	282 (72.5%)	325 (85.5%)		270 (81.8%)	
Presence	162 (21.1%)	107 (27.5%)	55 (14.5%)	<0.001	60 (18.2%)	0.275
Location						
Middle/Lower	516 (67.1%)	311 (79.9%)	205 (53.9%)		235 (71.2%)	
Upper	253 (32.9%)	78 (20.1%)	175 (46.1%)	<0.001	95 (28.8%)	0.179
Nodular composition						
Mixed cystic and solid	7 (0.9%)	4 (1.0%)	3 (0.8%)		5 (1.5%)	
Solid	762 (99.1%)	385 (99.0%)	377 (99.2%)	0.727	325 (98.5%)	0.376
Echogenicity						
Hyperechoic or isoechoic	25 (3.3%)	16 (4.1%)	9 (2.4%)		12 (3.6%)	
Hyperechoic	731 (95.1%)	365 (93.8%)	366 (96.3%)		317 (96.1%)	
Very hypoechoic	13 (1.7%)	8 (2.1%)	5 (1.3%)	0.280	1 (0.3%)	0.164
Shape						
A/T ≤1	273 (35.5%)	108 (27.8%)	165 (43.4%)		128 (38.8%)	
A/T >1	496 (64.5%)	281 (72.2%)	215 (56.6%)	<0.001	202 (61.2%)	0.299
Margin						
Smooth	487 (63.3%)	222 (57.1%)	265 (69.7%)		210 (63.6%)	
Lobulated or irregular	178 (23.1%)	97 (24.9%)	81 (21.3%)		80 (24.2%)	
ETE	104 (13.5%)	70 (18.0%)	34 (8.9%)	<0.001	40 (12.1%)	0.791
Echogenic foci						
None/large comet-tail artifacts	234 (30.4%)	87 (22.4%)	147 (38.7%)		104 (31.5%)	
Macrocalcifications	43 (5.6%)	24 (6.2%)	19 (5.0%)		19 (5.8%)	
Peripheral calcifications	7 (0.9%)	5 (1.3%)	2 (0.5%)		2 (0.6%)	
Punctate echogenic foci	485 (63.1%)	273 (70.2%)	212 (55.8%)	<0.001	205 (62.1%)	0.938
ETE detected during surgery						
Absence	628 (81.7%)	275 (70.7%)	353 (92.9%)		284 (86.1%)	
Presence	141 (18.3%)	114 (29.3%)	27 (7.1%)	<0.001	46 (13.9%)	0.075
LLNM						
Absence	703 (91.4%)	337 (86.6%)	366 (96.3%)		306 (92.7%)	
Presence	66 (8.6%)	52 (13.4%)	14 (3.7%)	<0.001	24 (7.3%)	0.468

PTC, papillary thyroid carcinoma; Y, year; BMI, body mass index; CLT, chronic lymphocytic thyroiditis; A/T, aspect ratio (height divided by width on transverse views); ETE, extrathyroidal extension; CLNM, central lymph node metastasis; LLNM, lateral lymph node metastasis.

P value < 0.05 indicates a significant difference between CLNM+ and CLNM− group in the training set.

P value* < 0.05 indicates a significant difference between training and validation sets.

In the training set, CLNM were observed in 389 (50.6%) cases. A significant difference was found in gender between CLNM-positive and CLNM-negative patients; 38.8% of males and 61.2% of females were CLNM-positive patients (*P*<0.001). Sonographic features, such as shape, margin and echogenic foci were all associated with CLNM. Moreover, CLNM presented the significant association with tumor size, the number of foci, bilaterality, location, ETE detected during surgery and LLNM (all *P*<0.05).

### Feature selection

We used the LASSO Logistic regression method to further select the optimal predictive features from the above characteristics. The optimal set of features that were most relevant to the prediction of CLNM included the following 13 features: sex, age, CLT, echogenicity, echogenic foci, shape, margin, size, location, bilaterality, number, ETE detected during surgery, LLNM (**Figure 1**).

### Predictive performance of ML-based models

**Figure 2:**
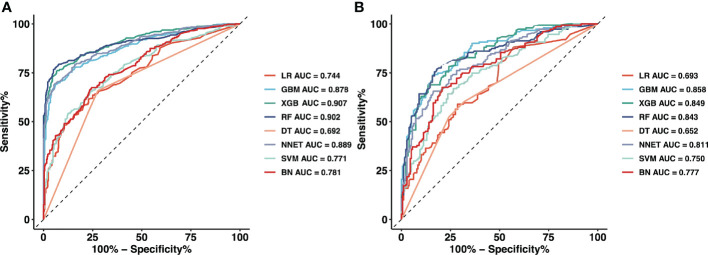
The mixed ROC curves of the eight machine learning models for prediction of CLNM. **(A)** The mixed ROC curves in the training cohort; **(B)** The mixed ROC curves in the validation cohort. *ROC*, receiver operating characteristic; *CLNM*, Central lymph node metastasis; *LR*, Logistic Regression; *GBM*, Gradient Boosting Machine; *XGB*, Extreme Gradient Boosting; *RF*, Random Forest; *DT*, Decision Tree; *NNET*, Neural Network; *SVM*, Support Vector Machine; *BN*, Bayesian Network.

**Figure 2** and **Table 2** show the predictive performance of ML-based models. In the training set, the AUCs of LR, GBM, XGB, RF, DT, NNET, SVM and BN were 0.744, 0.878, 0.907, 0.902, 0.692, 0.889, 0.771 and 0.781, respectively (**Figure 2** **A**). In the validation set, the AUCs of LR, GBM, XGB, RF, DT, NNET, SVM and BN were 0.693, 0.858, 0.849, 0.843, 0.652, 0.811, 0.750 and 0.777, respectively (**Figure 2** **B**). In the training cohort, the XGB model performed the best, followed by RF, NNET, and GBM. However, the sensitivity and specificity of RF were higher than that of XGB. All ML-based models except DT (AUC=0.777) and SVM (AUC=0.824) were better than the conventional method, LR (AUC=0.837). Apart from the DT, All ML-based models were better than the conventional method (LR).

**Table 2:** Predictive performance comparison of the eight types of machine learning algorithms in the training and validation dataset.

Methods	Training dataset			Validation dataset		
	AUC	Sensitivity	Specificity	AUC	Sensitivity	Specificity
LR	0.744	0.615	0.771	0.693	0.881	0.433
GBM	0.878	0.692	0.937	0.858	0.742	0.837
XGB	0.907	0.762	0.934	0.849	0.682	0.865
RF	0.902	0.767	0.950	0.843	0.795	0.798
DT	0.692	0.659	0.724	0.652	0.603	0.680
NNET	0.889	0.692	0.945	0.811	0.656	0.837
SVM	0.771	0.541	0.876	0.750	0.642	0.764
BN	0.781	0.674	0.755	0.777	0.675	0.792

AUC, the area under the curve; LR, logistic regression; GBM, gradient boosting machine; XGB, extreme gradient boosting; RF, random forest; DT, decision tree; NNET, neural network; SVM, support vector machine; BN, Bayesian network.

**Figure 3:**
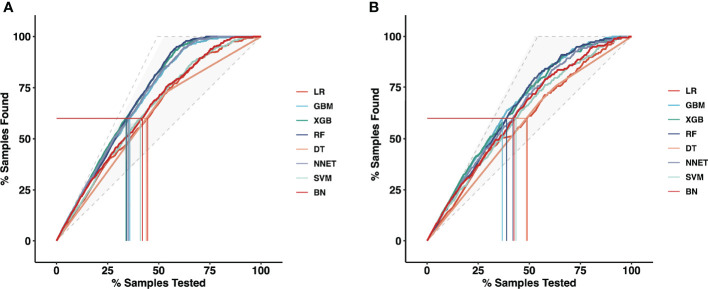
The mixed Lift curves of the eight machine learning models for prediction of CLNM. The drawing process of the Lift curve is similar to the ROC curve, the difference is that the Lift value and the robust plane pose change in opposite directions, forming the opposite form of the Lift curve and the ROC curve. **(A)** The mixed Lift curves in the training cohort; **(B)** The mixed Lift curves in the validation cohort. *CLNM*, Central lymph node metastasis; *ROC*, receiver operating characteristic; *LR*, Logistic Regression; *GBM*, Gradient Boosting Machine; *XGB*, Extreme Gradient Boosting; *RF*, Random Forest; *DT*, Decision Tree; *NNET*, Neural Network; *SVM*, Support Vector Machine; *BN*, Bayesian Network.

Moreover, the mixed Lift curves of the eight ML models were applied in the training and validation set (**Figure 3**). Different from the ROC curve, the Lift curve takes into account the accuracy of the classifier: the ratio of the number of positive classes obtained with the classifier to the number of positive classes obtained randomly without the classifier. XGB achieves the best diagnostic performance among the current mix Lift curves, followed by RF, NNET and GBM.

### Clinical usefulness of ML-based models

**Figure 4:**
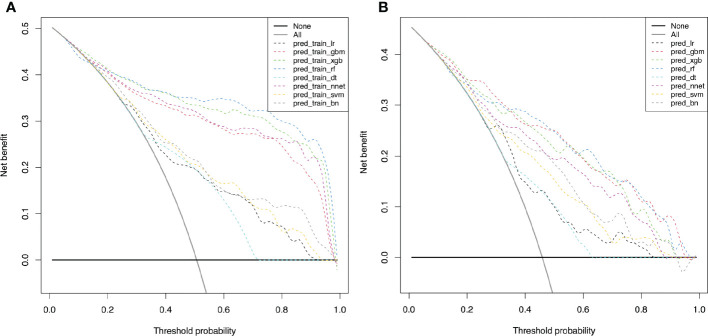
Decision curve for predictive models based on machine learning models for prediction of CLNM. **(A)** The decision curve in the training cohort; **(B)** The decision curve in the validation cohort. *CLNM*, Central lymph node metastasis; *LR*, Logistic Regression; *GBM*, Gradient Boosting Machine; *XGB*, Extreme Gradient Boosting; *RF*, Random Forest; *DT*, Decision Tree; *NNET*, Neural Network; *SVM*, Support Vector Machine; *BN*, Bayesian Network.

DCA was further used to evaluate the clinical values of these models (**Figures 4**). The solid black line (None line) represents the net benefit is zero when none of patients receive CND, assuming that all patients have no positive nodes in the central compartment. On the contrary, the solid grey line (All line) represents the net benefits at the time when all patients have CLNM and receive CND. Most of these models presented better net benefits than two control models that were represented by solid black and solid grey lines. Four models (RF, XGB, NNET, and GBM) performed significantly better than the others at most of threshold points. In the training cohort, RF performed significantly better than the others at most of threshold points, followed by XGB (**Figures 4** **A**). In the validation cohort, GBM performed the best at the threshold range of 0.2 to 0.4, but sharply decreased at the threshold range of 0.4 to 0.7. RF performed the best at the threshold range of 0.4 to 0.7, but sharply decreased at the threshold range of 0.8 to 0.9 (**Figures 4** **B**).

### Relative importance of variables in ML-based models

**Figure 5:**
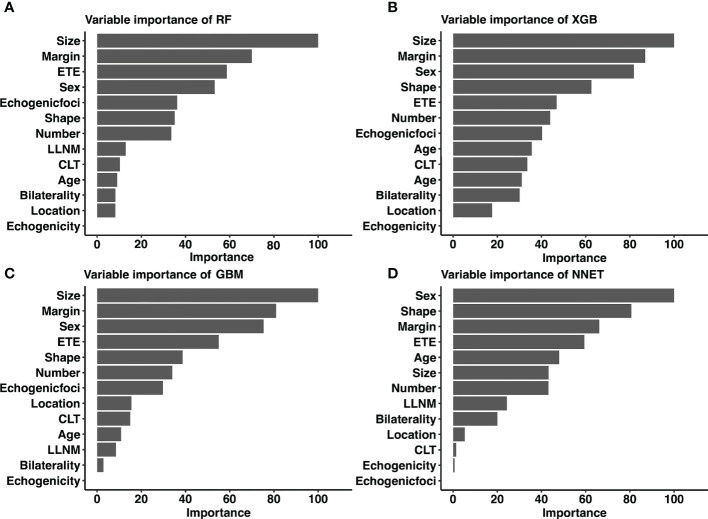
Relative importance ranking of each input variable for prediction of CLNM in the machine learning models. **(A)** RF model; **(B)** XGB model; **(C)** GBM model; **(D)** NNET model. *CLNM*, Central lymph node metastasis; *ETE*, extrathyroidal extension; *LLNM*, lateral lymph node metastasis; *CLT*, chronic lymphocytic thyroiditis; *RF*, Random Forest; *XGB*, Extreme Gradient Boosting; *GBM*, Gradient Boosting Machine; *NNET*, Neural Network.

Considering favorable AUCs and clinical benefits based on the DCA, we selected XGB, RF, NNET, and GBM as the models with the most potential for predicting CLNM in cN0 PTC patients. By the feature selection approach, we ranked 13 variables based on their predictive importance in each potential model. The ranks of each variable in different models were described in **Figure 5**. Size, margin and sex were considered as the relatively important variables for predicting CLNM in the vast majority of models.

**Figure 6:**
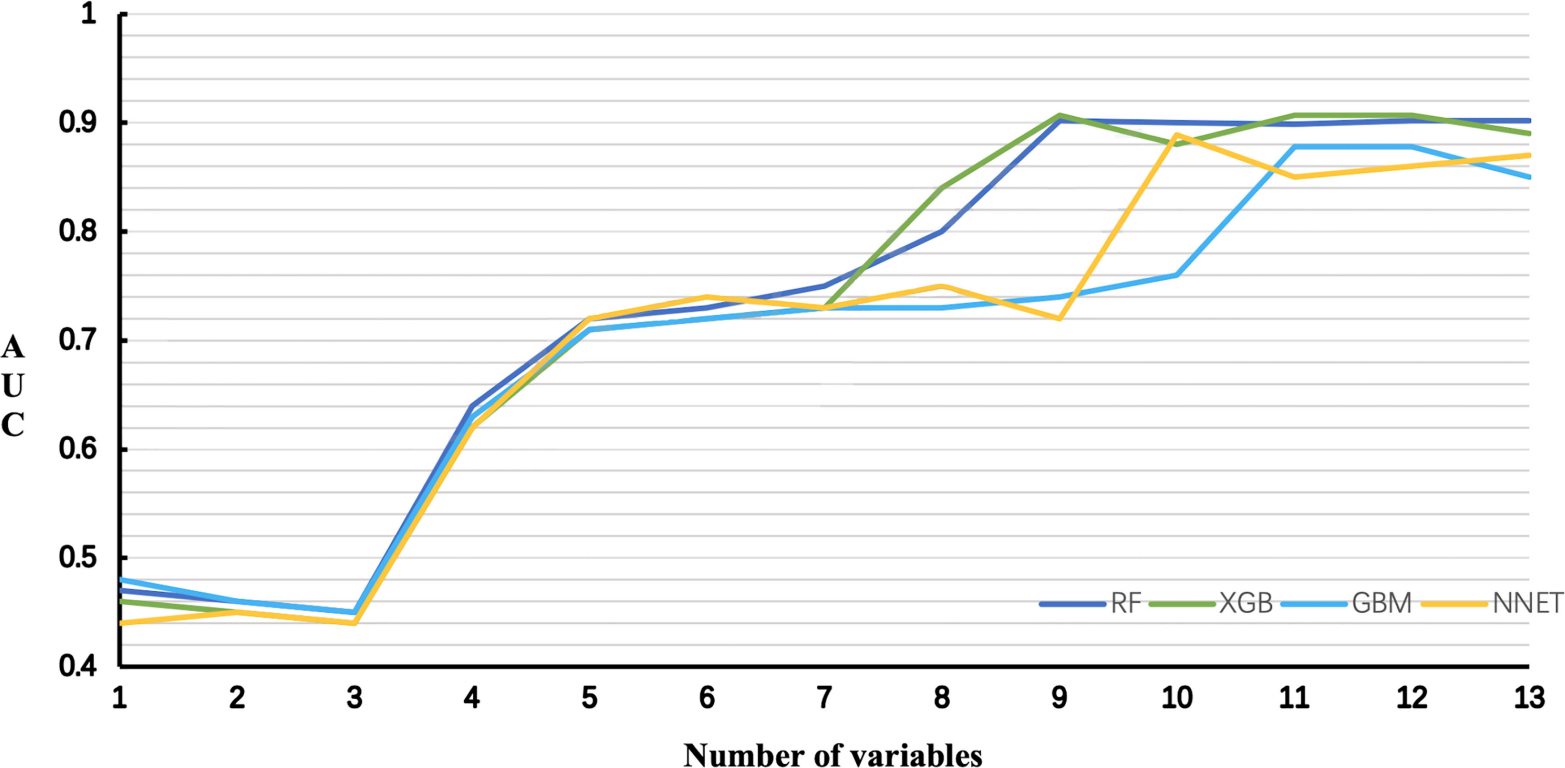
Predictive performance of the RF, XGB, GBM, NNET models with different numbers of variables. *RF*, Random Forest; *XGB*, Extreme Gradient Boosting; *GBM*, Gradient Boosting Machine; *NNET*, Neural Network.

The AUCs of RF and XGB reached the highest when 9 variables were introduced (**Figure 6**). As for GBM and NNET reached the highest when 11 and 10 variables were introduced (**Figure 6**).

Taking into account the sensitivity, specificity, AUC, Lift curve and DCA of the model, we chose RF as the best predictive model in this study. The nine top-rank variables were identified to construct the best predictive model, including size, margin, ETE, sex, echogenic foci, shape, number, LLNM and CLT.

## Discussion

At present, some risk factors related to CLNM have been identified, such as tumor differentiation, gene types, etc (26). However, these risk factors are only available after surgery, and they can not provide important information for the preoperative therapeutic decisions. In addition, due to the air in the trachea and the complex structure of the sternum and clavicle, ultrasound is difficult to detect CLNM accurately (9, 10). Combined with the above considerations, we incorporated some variables that can be obtained before and during the operation to build non-invasive and valuable ML models to predict CLNM.

The advantage of ML algorithms is their ability to automatically learn from input data and identify patterns and trends in these data. At present, several studies have used ML for the differential diagnosis of benign and malignant thyroid nodules (27, 28). In addition, ML has also been used to predict LNM in some other malignant tumors, such as breast cancer and osteosarcoma, etc (29, 30). However, there is little research on the application of ML model predicting LNM in PTC patients. Lee et al. (31) applied clinical records for 804 consecutive patients to develop a computer-aided diagnosis system to identify and differentiate metastatic lymph nodes in thyroid cancer. However, the specificity of the model is relatively low, and the screening results should also be verified by experienced physicians. In addition, they used only one ML model and did not compare the performance of multiple ML models in distinguishing metastatic lymph nodes in patients with thyroid cancer. The predictive performance of different machine learning algorithms is different. We adopted the eight most important ML algorithms to construct the CLNM prediction model, and selected an optimal prediction model from these to ensure the effectiveness.

We first used the LASSO Logistic regression method to exclude four variables (BMI, nodular composition, BRAF V600E mutation and diabetes) that would affect the fitting. And then, modeling the training set of 769 cases of data showed that four excellent models (XGB, RF, NNET, and GBM) performed better in both the ROC analysis and mix Lift curves. The AUC of XGB was slightly higher than RF. However, the RF model performed significantly better than the XGB model at most of threshold points according to DCA. Therefore, we choose RF as the best predictive model in this study to distinguish CLNM from non-CLNM. The structure of RF is simple. It is operated by constructing a large number of decision trees and outputting classes as a single tree (classification) or average prediction (regression) model. Compared with similar methods, RF is more efficient. From a computational point of view, RF has the advantage of handling both regression and classification problems. High dimensional problems can also be directly handled through RF (32). From a statistical point of view, RF has the following characteristics, that is, the priority of characteristics, different weight coefficients fall into different categories, and illustration and unsupervised learning ability (33). According to previous meta-analysis of metastatic lymph node studies, computed tomography (CT) demonstrated a pooled sensitivity of 57% and a specificity of 85% in detecting CLNM, and ultrasound demonstrated a pooled sensitivity of 38% and a specificity of 91%. Combined CT/ultrasound demonstrated a pooled sensitivity of 69% and a specificity of 81% (34). When we compared the diagnostic performance of the RF model with that in the meta-analysis, our RF model achieved better sensitivity (0.767) and specificity (0.950).

The connection between variables and results in most ML-based models is invisible. By using classifier-specific estimators, we got the predictive importance of variables in each model (**Figure 5**). Therefore, the nine top-rank variables were identified to be the most important risk factors for CLNM in the RF model: size, margin, ETE, sex, echogenic foci, shape, number, LLNM and CLT. It is important to note that size was the largest contributor to scores in most models (including RF, XGB and GBM), which was consistent with other reports (35). Tumor size is widely used in several staging systems, including the American Joint Committee on Cancer staging system. And larger tumor size was associated with more aggressive features in PTC (35). Based on the combined RF model incorporating clinicopathological and sonographic features, for patients with several risk factors of CLNM, prophylactic CND is strongly recommended to reduce recurrence rates. In addition, it is recommended that experienced surgeons perform detailed operations on these high-risk patients, during which carbon nanoparticles suspension injection can be used to prevent miss of small metastatic lymph nodes. Otherwise, prophylactic CND should be avoided to reduce complications of parathyroid glands and recurrent laryngeal nerve. In addition, for high-risk patients who did not undergo CND, these patients should be followed up more closely after surgery to increase vigilance against occult CLNM.

The strength of this research lies in the innovation of technology and method. Although CLNM is predicted by filtering the best model from eight ML methods, there are also limitations. The first is to retrospectively study the inherent limitations in the design. This study is a single-center retrospective study, our results may be biased and lack generalizability and robustness assessments. Second, the patients who participated in our study were the local population of China, most of whom were women. Residual confounding variables of unmeasurable factors such as race and region cannot be ruled out. Prospective multi-center clinical trials need to be carried out in subsequent studies to obtain more objective conclusions. Third, the criteria used to evaluate ultrasound characteristics were subjective. Nevertheless, the consistency between the observers of each feature in this study was very good. Last, most of algorithms are invisible to users. In the future study, by using the web-based calculator which established based on our prediction model, we can apply our findings to other population.

In conclusion, by incorporating clinicopathological and sonographic characteristics, we developed ML-based models, suggesting that this non-invasive method can be applied to facilitate individualized prediction of occult CLNM in cN0 central neck PTC patients. The status of lymph nodes is evaluated through the RF model, and it is recommended to perform prophylactic CND for high-risk patients.

## Data availability statement

The raw data supporting the conclusions of this article will be made available by the authors, without undue reservation.

## Ethics statement

Informed consent was obtained from all individual participants included in the study. Written informed consent was obtained from the individual(s) for the publication of any potentially identifiable images or data included in this article.

## Author contributions

J-WF and L-ZH: Writing - Original Draft, Software, Data Curation. S-YL: Validation, Formal analysis, Data Curation. FW: Conceptualization. JY and G-FQ: Validation, Investigation. YJ: Writing - Review and Editing, Visualization, Supervision. All authors contributed to the article and approved the submitted version.

## Acknowledgments

Lei Qin, the English language editor, was responsible for correcting language and grammar issues.

## Conflict of interest

The authors declare that the research was conducted in the absence of any commercial or financial relationships that could be construed as a potential conflict of interest.

## Publisher’s note

All claims expressed in this article are solely those of the authors and do not necessarily represent those of their affiliated organizations, or those of the publisher, the editors and the reviewers. Any product that may be evaluated in this article, or claim that may be made by its manufacturer, is not guaranteed or endorsed by the publisher.
